# MIRU-VNTR Genotyping of *Mycobacterium tuberculosis* Strains Using QIAxcel Technology: A Multicentre Evaluation Study

**DOI:** 10.1371/journal.pone.0149435

**Published:** 2016-03-03

**Authors:** Vladyslav Nikolayevskyy, Alberto Trovato, Agnieszka Broda, Emanuele Borroni, Daniela Cirillo, Francis Drobniewski

**Affiliations:** 1 Infectious Diseases, Imperial College London, London, United Kingdom; 2 Division of Infectious Disease, Immunology and Transplantation, IRCCS San Raffaele Scientific Institute, Milan, Italy; Fudan University, CHINA

## Abstract

**Background:**

Molecular genotyping of *M*.*tuberculosis* is an important laboratory tool in the context of emerging drug resistant TB. The standard 24-loci MIRU-VNTR typing includes PCR amplification followed by the detection and sizing of PCR fragments using capillary electrophoresis on automated sequencers or using agarose gels. The QIAxcel Advanced system might offer a cost-effective medium-throughput alternative.

**Methods:**

Performance characteristics of the QIAxcel Advanced platform for the standard 24 VNTR loci panel was evaluated at two centres on a total of 140 DNA specimens using automated capillary electrophoresis as a reference method. Additionally 4 hypervariable MIRU-VNTR loci were evaluated on 53 crude DNA extracts. The sizing accuracy, interlaboratory reproducibility and overall instrument’s performance were assessed during the study.

**Results:**

An overall concordance with the reference method was high reaching 98.5% and 97.6% for diluted genomic and crude DNA extracts respectively. 91.4% of all discrepancies were observed in fragments longer than 700bp. The concordance for hypervariable loci was lower except for locus 4120 (96.2%). The interlaboratory reproducibility agreement rates were 98.9% and 91.3% for standard and hypervariable loci, respectively. Overall performance of the QIAxcel platform for *M*.*tuberculosis* genotyping using a panel of standard loci is comparable to that of established methods for PCR fragments up to 700bp. Inaccuracies in sizing of longer fragments could be resolved through using in-house size markers or introduction of offset values. To conclude, the QiaXcel system could be considered an effective alternative to existing methods in smaller reference and regional laboratories offering good performance and shorter turnaround times.

## Background

The development and introduction of molecular genotyping methods for *M*. *tuberculosis* has significantly improved our knowledge of TB transmission, bacterial phylogeny and evolution [1]. PCR-based genotyping methods including multilocus MIRU-VNTR require small amounts of non-purified DNA and produce results in a digital format enabling portability of results and creation of national and international databases for routine and research purposes [2, 3]. More recently, whole genome next generation sequencing technologies became available providing even higher discriminatory power [4].

MIRU-VNTR typing utilizes variations in repetitive sequences which are not under selection pressure and evolve relatively rapidly making them suitable for prospective molecular epidemiological investigations and surveillance purposes [3, 5]. MIRU-VNTR genotyping using a standardised set of 24 loci has become an international standard and is currently in use for routine *M*. *tuberculosis* genotyping in many European countries and globally [6]. The discriminatory power of MIRU-VNTR typing generally depends on the number and set of loci used and could be further improved by inclusion of hypervariable loci especially in settings where highly homogenous genotypes (East Asian lineage eg Beijing) prevail [7, 8].

National or subnational TB molecular surveillance systems based on multilocus VNTR typing have been established in several EU countries and pilot international databases developed [9]. Genotyping is currently recognized as an essential tool for tracing outbreaks, transmission and surveillance purposes to facilitate public health interventions and study *M*. *tuberculosis* population structure in diagnostic and research contexts. Multicenter proficiency studies conducted in 2009 and 2010 demonstrated improvements in both inter- and intralaboratory reproducibility of genotyping results also highlighting specific issues including lack of standardization and poorer performance associated with the PCR fragments separation using manual electrophoresis [9, 10].

Both commercial and in-house methods are currently in use for VNTR typing in routine laboratory settings; the separation and sizing of the PCR fragments could be done on automated sequencers or manually using agarose gels. The latter option, generally being the cheapest one, is used in many laboratories in the EU and elsewhere despite the reported reproducibility issues [9, 10] and low throughput.

Cost and infrastructure implications may limit access of TB diagnostic laboratories to VNTR genotyping especially in medium- and high TB and drug resistant TB burden areas compromising their ability to perform real-time molecular epidemiology investigations. The QiaXcel platform developed by Qiagen offers an affordable medium-throughput alternative to the methods based on the separation of fluorescent-labelled PCR fragments using capillary electrophoresis [11]. In a recent study performed on a convenience sample of 82 *M*. *tuberculosis* isolates using 15- and 24 loci standard VNTR panels, overall concordance rate with the reference method was 98.9% demonstrating potential suitability of the system for routine *M*. *tuberculosis* genotyping [12].

In our multicentre study we aimed to assess performance the QiaXcel platform for TB genotyping using the most reproducible hypervariable loci [7] and establish its interlaboratory reproducibility using a standardized well-characterized panel of *M*. *tuberculosis* strains.

## Materials and Methods

### Study design and test panels

The study was conducted at two centres, Imperial College (London, UK) and the San Raffaele Scientific Institute (Milan, Italy), where a total of 140 DNA specimens were tested using the QiaXcel methodology and the reference method. London and Milan centres tested 100 and 140 DNA specimens grouped into three panels as follows:

- Panel 1 comprised 60 purified genomic *M*. *tuberculosis* DNA specimens (at 1 ng/μl concentration). DNA samples in this panel were identical at both sites and were used for evaluation of interlaboratory reproducibility for standard 24 VNTR loci.
- Panels 2 (London) and 3 (Milan) each included 40 crude *M*. *tuberculosis* DNA extracts and were used for the assessment of the performance of QiaXcel system for genotyping using hypervariable loci, for further adjustment of the system PCR fragments separation parameters for specific loci, and for evaluation of interlaboratory reproducibility for hypervariable loci (Panel 2 only). Panels comprised strains from various genetic lineages including Beijing family strains (6 and 13 extracts respectively).

All panels included *M*. *tuberculosis* strains from various lineages; panels 2 and 3 were specifically enriched with Beijing family strains to test the performance of the system for highly conserved genotyped using hypervariable loci. Research was carried out on the *M*. *tuberculosis* DNA specimens stored in the laboratories as a part of past proficiency testing schemes (genomic DNAs) and past research projects (crude DNAs) and did not involve any human subjects, specimens or tissues. DNA specimens were fully anonymised, any information obtained could not be traced back to the original *M*. *tuberculosis* cultures or patients and no patient information was collected/analysed therefore ethical approval was not sought.

### VNTR loci and PCR

In the current study, we evaluated the performance of the system using a standardised set of 24 VNTR loci and four hypervariable loci (VNTR 3232, 1982, 3820, and 4120). PCR fragments for all VNTR loci were generated individually in monoplex PCRs using sets of primers and conditions as outlined in previous publications and manuals [6, 7, 13]. For the analysis on QiaXcel system, unlabelled primers were used. PCR was performed in a volume of 20μl and upon completion of the amplification the PCR plate was loaded onto the QiaXcel analyser for the fragment separation and allele calling. Negative controls (with purified water instead of DNA) and size marker were included into each run.

### Reference methods

In London, in-house PCR with fluorescent-labelled primers followed by fragment analysis using an automated capillary sequencer Beckman Coulter CEQ8000 was used as the reference method. PCR, fragment separation, sizing and allele calling was done as described previously [14]. Labelled primer sequences were identical to unlabelled ones.

In Milan, reference values were obtained by using the 24 MIRU-VNTR typing kit (Genoscreen, Lille, France), made up of 6 different multiplex (quadruplex) PCRs. The run was performed by using an automatic sequencer, 3730 DNA analyzer (Applied Biosystems, California, USA), and then analyzed by proprietary GeneMapper® v3.7 software. Hypervariable loci were characterized by a simplex PCR followed by the PCR products analysis on 3% agarose gel.

In both centers, numbers of repeats in individual loci were entered into Excel spreadsheets and concordance with QiaXcel results was analysed.

### PCR fragment separation and allele calling using QiaXcel

PCR fragment separation on QiaXcel system was performed according to the manufacturer’s recommendations as described previously [12]. Briefly, undiluted PCR products in a 96-well plate were loaded onto the system, and PCR fragments separation was performed for 1,700 s at 2 kV with an alignment marker 15 to 3,000 bp and size marker 100 to 2,500 bp. Fragment sizing and allele calling was performed automatically by the QiaXcel software using expected PCR fragment sizes [7, 13]; tolerance for individual loci was calculated as described previously [12]. Test results (number of repeats in individual loci) were exported and entered into Excel spreadsheets for further analysis. If a PCR product was missing/not detected on the QiaXcel system, PCR and separation was repeated once and this result was considered final and entered in the database. In case of multiple peaks on QiaXcel system, histograms were visually examined by an operator and allele assignation was made based on recommendations [13].

### Performance and interlaboratory reproducibility assessment

The performance and accuracy of the QiaXcel system was assessed through calculation of overall and individual 28 loci concordance rates between the reference and QiaXcel methods results. To evaluate interlaboratory reproducibility, agreement rates between the QiaXcel results obtained at the two laboratories for the isolates included in the Panel 1 and Panel 2 were calculated.

## Results

### QiaXcel test performance characteristics

#### Standard loci

**Fig 1:**
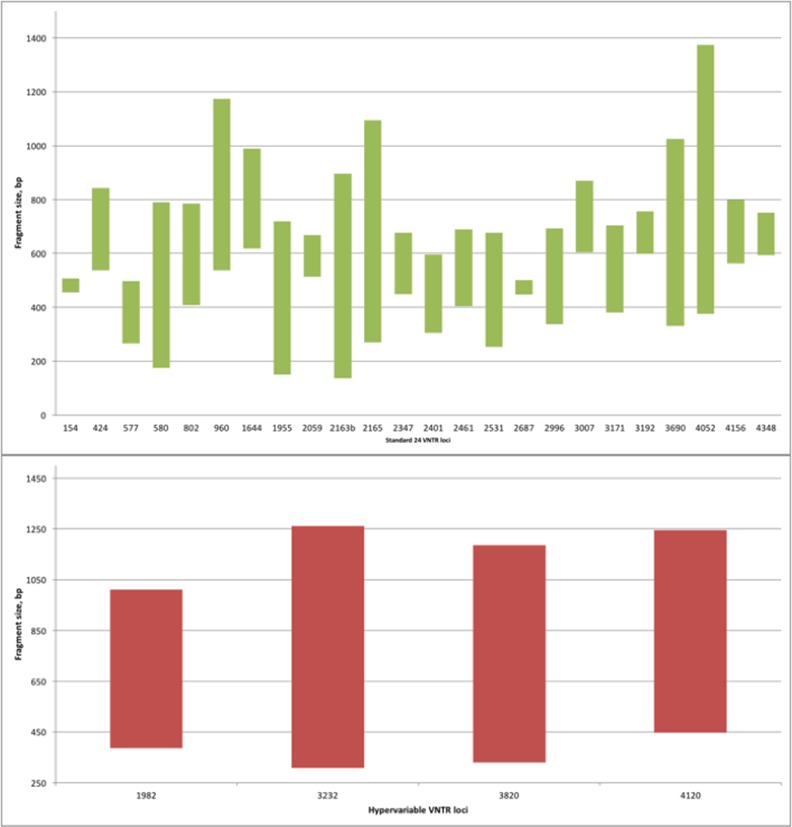
Sizes of PCR fragments determined by the QiaXcel system. A–Standard loci. B–Hypervariable loci.

Performance of the assay for a standard 24 VNTR loci was assessed at both centres using two panels of *M*. *tuberculosis* DNA specimens (diluted genomic DNA and crude DNA extracts). In total, 100 DNA samples were tested with a total of 2,400 assays run at each centre. PCR fragment sizes as determined by the reference methods varied significantly depending on the number of copies within specific VNTR loci with the shortest (136 bp) and longest (1374 bp) fragments seen in loci 2163b and 4052, respectively (Fig 1)

Overall readability and agreement rates between QiaXcel results and reference values were high. Readability was analysed separately for the panels comprising genomic and crude DNA; in the latter panel the PCR failure rate was greater (4.6%, N = 44) compared to genomic DNA (0.4%, N = 6). The greatest number of PCR failures was observed in panel 2 formed of 40 non-preselected (routine) crude DNA extracts analysed in London; nearly all PCR failures (N = 31) were confined to two isolates from Panel 2 in which just over half of all loci were amplified which may indicate problems with the quality of these two particular DNA extracts. There were no systematic differences in readability of different VNTR loci; no PCR failures were seen in 18 and 7 loci in genomic and crude DNA extract panels, respectively.

No discrepancies between QiaXcel results and reference values were observed in 14 loci (Table 1). For the other ten loci agreement rates varied from 59.0% (locus 4052, crude DNA extracts) to 99.0% with disagreements seen more commonly in crude DNA extracts. In all but one locus (VNTR4052) concordance rates were consistently over 95%. In locus 4052, poorer agreement rates (59.0% and 80.8% for crude and genomic DNA, respectively) were associated with the greater molecular weights of PCR fragments (Table 2).

**Table 1:** Combined agreement rates between QiaXcel and reference methods.

VNTR locus		Concordance, %	
Position	Alias	Panel 1 (diluted gDNA)	Panels 2 and 3 (crude DNA)
154	MIRU2	100	100
424		100	98.8
577	ETR-C	100	100
580	MIRU4	100	98.8
802	MIRU40	100	100
960	MIRU10	100	100
1644	MIRU16	98.3	95.0
1955		95.0	100
2059	MIRU20	100	100
2163b		95.8	97.4
2165	ETR-A	96.7	100
2347		100	100
2401		100	100
2461	ETR-B	100	100
2531	MIRU23	100	100
2687	MIRU24	100	100
2996	MIRU26	100	100
3007	MIRU27	100	98.8
3171		100	98.8
3192	MIRU31	100	100
3690		97.5	95.0
4052		80.8	59.0
4156		100	100
4348	MIRU39	100	100

**Table 2:** Discrepant results for standard loci across two centres.

Loci	No of discrepantresults	Qiaxcel	Reference	
		No of repeats	PCR fragment size bp (range)	No of repeats
424	1	7	894	6
580	1	9	868	8
1644	6	4, 7, 9	777–1042	3, 6, 8
1955	6	12	776	11
2163b	7	1, 2, 13, Multiple peaks	146–974	4, 6, 2/3, 12,1, 8,12
2165	4	10,13	945–1170	9,12
3007	1	8	922	7
3171	1	7/8	704/758	7
3690	7	9, >15, 7, 9, 3, 6, 3/5	446- >1142	8, 13, 2, 8, 8, 10, 5
4052	55	7, 8, 9, 10, 11, 12	1019–1509	6, 7, 8, 9, 10, 11

Discrepancies between the results obtained using QiaXcel and reference methods are summarised in Table 2. Discrepant results almost exclusively (74/81, 91.4%) were due to an overestimation of the PCR fragment sizes >700 bp and subsequent incorrect calculation of the number of repeats within respective loci seen on the QiaXcel system. In most cases, except in loci 3690 and 2163b, the number of copies was overestimated by one reflecting an incorrect sizing of larger PCR fragments due to observed lengths exceeding tolerance values pre-set on the system. In locus 2163b in three specimens (Panels 1 and 2 in London) multiple peaks and/or “bell-shaped” distribution of peaks on histogram were observed which made unambiguous sizing and calculation of number of repeats challenging even using visual examination of histograms. Remaining discrepant results (n = 4) were sporadic and not confined to any specific loci.

#### Hypervariable loci

Performance of the QiaXcel for hypervariable VNTR loci (1982; 3232; 3820; and 4320) was tested using crude extracts included in Panel 2 (London) and Beijing isolates (N = 13) from Panel 3 (Milan). PCR fragment sizes for hypervariable loci as detected by the reference method are presented in Fig 1.

Within this group, the PCR failure rate varied between loci substantially. It was higher for loci 1982 and 3232 (15.4% and 7.8%, respectively) while in locus 4120 all results were readable. Agreement between QiaXcel and reference values for hypervariable loci also varied being low for three loci (34.7%, 59.4%, and 61.5% for loci 3232, 1982, and 3820, respectively) and high for locus 4120 (96.2%). Discrepancies between the results obtained using QiaXcel and reference methods for hypervariable loci are summarised in Table 3.

**Table 3:** Discrepant results for hypervariable loci.

Loci	No of discrepantresults	Qiaxcel	Reference		Agreement rates	
		No of repeats	PCR fragment size (range)	No of repeats	Beijing	Non-Beijing
1982	16	2, 10, 1/9, 9, 1, 8, 9	308–1010	10, 9, 8, 8, 9, 9, <11	59.4	71.4
3232	24	1/3, 15, 2, 6, 3, 7, 5, 1/2/3, 1/2, 13, 13, 22, 14, 14, 13	309–1037	16, 13, 10, 5, 7, 5, 3, 14, 18, 14, 16, >23, 16, 17, 15	34.7	77.4
3820	10	15, 13	1014–1128	14, 12	61.5	100.0
4120	1	16	>1188	15	96.2	100.0

**Fig 2:**
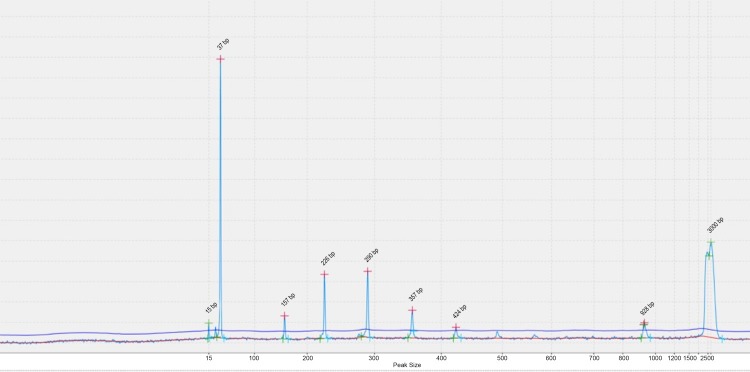
Example of an incorrect Locus 3232 peak calling on the QiaXcel system.

Both PCR failures and discordant results were mainly confined to long PCR fragments exceeding 700 bp in size. Overestimation of PCR fragment sizes by 50–100 bp seen on the QiaXcel system led to incorrect assignation of allelic variant/number of repeats, specifically in loci 1982 and 3232. In five specimens with a larger number of repeats in loci 1982 and 3232 (9 and 15–18, respectively), stutter peaks (1–3 copies) were miscalled instead (Fig 2), and in three more cases no peaks were observed due to insufficient separation time. Notably, Beijing family strains known to have larger numbers of copies in loci 3232 and 1982 were the most problematic; for non-Beijing strains concordance rates for all hypervariable loci were significantly better being as high as 100% for loci 3820 and 4120 (Table 3).

### Interlaboratory reproducibility

**Fig 3:**
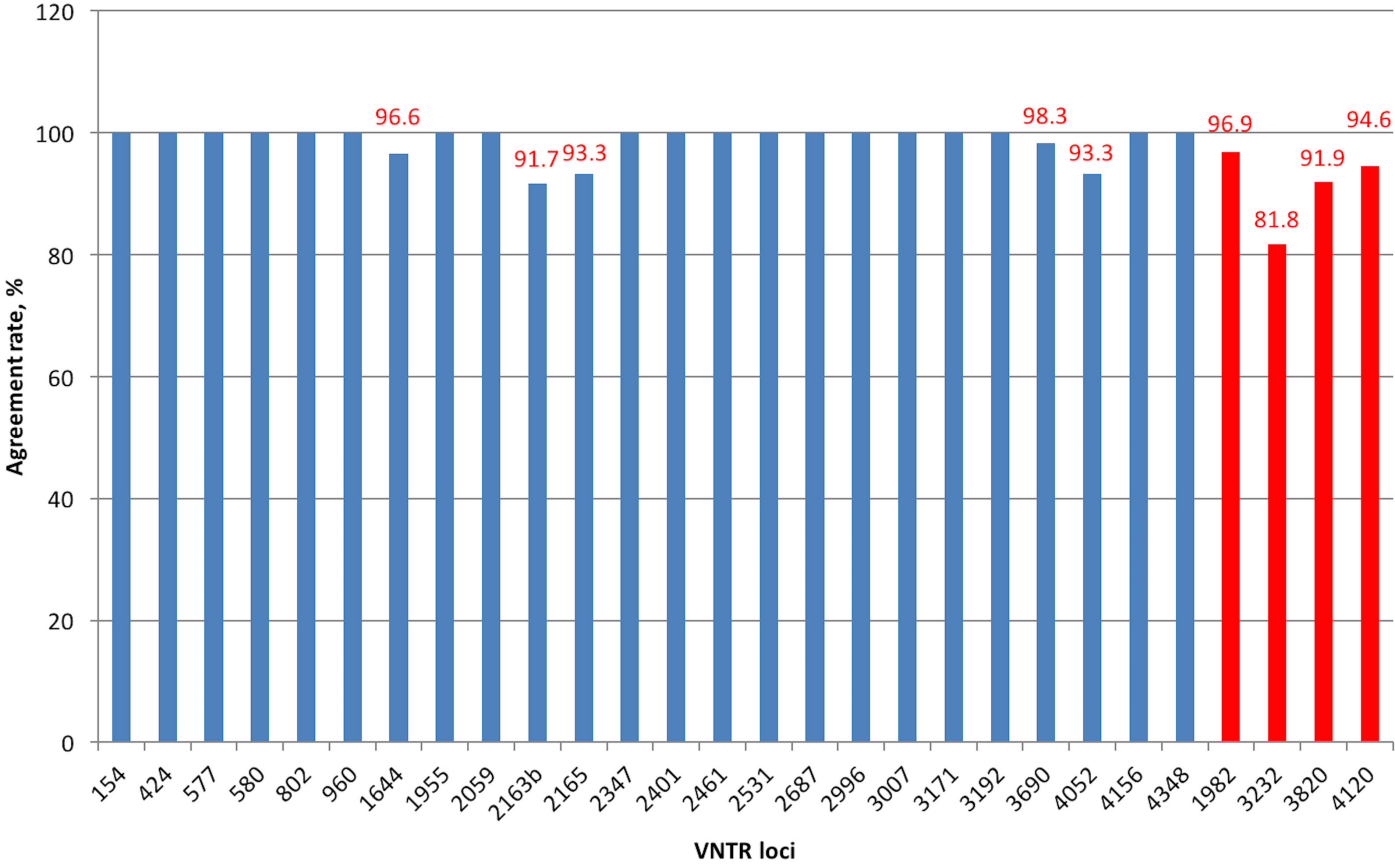
Interlaboratory agreement rates. Hypervariable loci shown in red colour. In cases where results from both laboratories were incorrect, these values were not included in discrepancies.

Interlaboratory reproducibility was assessed through comparing data generated at both laboratories using Panels 1 (for standard loci) and 2 (for hypervariable loci). Overall agreement rates were good (Fig 3) with the number of discrepancies (N = 28) comprising 0.9% of the total number of PCR assays.

Results between the two laboratories were fully concordant for 19 standard VNTR loci; for the other five standard loci and four hypervariable loci agreement rates varied from 91.7% to 98.3% and from 81.8% to 96.9%, respectively. Again, discordances were predominantly seen in specimens with relatively large numbers of repeats in specific VNTR loci with the greatest number of discordances seen in loci 2163b, 3232, 3820, 2165, and 4052. There were no major differences in interlaboratory reproducibility rates between standard and hypervariable loci.

## Discussion

In the current multicentre study performed at two European laboratories in Milan and London, we aimed to assess performance characteristics and establish interlaboratory reproducibility of the innovative QiaXcel system for the multilocus VNTR genotyping of *M*. *tuberculosis* using panels of standard and hypervariable loci. Overall performance characteristics of the system for standard loci were high with readability rates of 99.6% and 95.4% for the diluted genomic and crude DNA extracts, respectively. Agreement rates vs reference values for the PCR fragments sized 136–758 bp were excellent reaching 99.9% with the number of discordant results (n = 4) accounting for 2.2% of all discrepancies. Excellent interlaboratory agreement rates were observed in our study (99.1%) confirming reproducibility of the method and, importantly, potential portability of results between different laboratories both for standard and hypervariable loci. These findings are in agreement with earlier reports and demonstrate a potential applicability of the QiaXcel system in *M*. *tuberculosis* VNTR genotyping using both genomic and crude DNA extracts; it also highlighted persistent problems with higher molecular weight fragments reported in earlier studies [11, 12].

Results of our study suggest that differences in quality and concentrations of DNA specimens have only a minor impact on the performance of the system (Table 1). Accuracy depended primarily on the PCR fragment length regardless of the locus analysed being suboptimal for sizes >750 bp which translated into the poor performance of the system for a small number of both standard (4052) and hypervariable (1982, 3232, and 3820) loci where variants with a greater number of copies occur frequently. Problems with sizing of longer PCR fragments on capillary electrophoresis systems have been reported previously [15, 16]. Reasons for inaccurate sizing and subsequent incorrect assignation of allelic variants on capillary systems include anomalous migration of the DNA due to secondary structures, variations in migration rates under normal and denaturing conditions [13, 17] as well as suboptimal fragment separation parameters and hardware limitations. These problems could be both loci-specific and PCR fragment size-dependant and require optimization of both separation procedures and peak calling algorithms. Previously loci 4052, 1982, and 3232 have been found to be particularly problematic [15] which is in agreement with our study results.

Experimental determination of offset values for specific loci using reference strains with known numbers of VNTR copies in each locus and introduction of appropriate changes into the calling algorithms and reference tables may help to improve accuracy of PCR fragment sizing and eliminate errors in assignation of allelic variants [13, 15]. Modified calling tables with offset values for locus 4052 were tested in one of the study laboratories with excellent results (data not shown) significantly improving performance of the system. This approach has proven effective in earlier studies for other loci [15, 18, 19] and could be recommended for locus 4052 and other “difficult to type” loci. An alternative approach utilising allele-specific size markers for individual or groups of loci [11, 15] is currently used in commercial VNTR genotyping kits for capillary sequencers (eg Genoscreen, Lille, France) and proved effective for VNTR genotyping both on cultures and clinical specimens [7, 20]. Both approaches require a careful calibration of the system using reference *M*. *tuberculosis* and/or *M*. *bovis* BCG strains to ensure accuracy and reproducibility of results.

Other issues, including missing higher molecular weight peaks, could be resolved through adjustment of the separation parameters, including longer separation time and lower voltage. This would increase the resolution of the system and ensure effective separation of PCR fragments >1000 bp potentially eliminating problems with loci 4052, 1982, 3232, and 3820 providing the system has been calibrated to take offset values into account.

To conclude, the QiaXcel system could be considered an effective alternative to existing automated capillary electrophoresis sequences in smaller reference and regional laboratories with intermediate workload offering a performance superior to manual gel electrophoresis PCR fragment separation, shorter turnaround times and relative technical simplicity. Cost effectiveness of the system is yet to be evaluated in line with national and international policies on *M*. *tuberculosis* genotyping in given settings. Importantly, the system could be potentially used for automated PCR fragment separation and sizing in other typing technologies, including indel mapping and multilocus SNP-based typing [21, 22] which makes it a versatile tool for both routine epidemiological investigations and phylogenetic/evolutionary research. Existing problems with inaccurate sizing of larger DNA fragments in loci 4052, 3232, and 1982 could be addressed through adjustment of calling algorithms using either offset values or allelic size markers with prior calibration of the system.

## References

[1] Niemann S, Supply P. Diversity and evolution of Mycobacterium tuberculosis: moving to whole-genome-based approaches. Cold Spring Harbor perspectives in medicine. 2014 12;4(12):a021188 10.1101/cshperspect.a021188 25190252 PMC4292095

[2] Kato-Maeda M, Metcalfe JZ, Flores L. Genotyping of Mycobacterium tuberculosis: application in epidemiologic studies. Future Microbiol. 2011 2;6(2):203–16. Epub 2011/03/04. eng. 10.2217/fmb.10.165 21366420 PMC4296029

[3] Jagielski T, van Ingen J, Rastogi N, Dziadek J, Mazur PK, Bielecki J. Current methods in the molecular typing of Mycobacterium tuberculosis and other mycobacteria. Biomed Res Int. 2014;2014:645802 Pubmed Central PMCID: 3914561. 10.1155/2014/645802 24527454 PMC3914561

[4] Walker TM, Ip CL, Harrell RH, Evans JT, Kapatai G, Dedicoat MJ, et al Whole-genome sequencing to delineate Mycobacterium tuberculosis outbreaks: a retrospective observational study. The Lancet infectious diseases. 2013 2;13(2):137–46. Pubmed Central PMCID: 3556524. 10.1016/S1473-3099(12)70277-3 23158499 PMC3556524

[5] Crawford JT. Genotyping in contact investigations: a CDC perspective. The international journal of tuberculosis and lung disease: the official journal of the International Union against Tuberculosis and Lung Disease. 2003 12;7(12 Suppl 3):S453–7. . 14677837

[6] Supply P, Allix C, Lesjean S, Cardoso-Oelemann M, Rusch-Gerdes S, Willery E, et al Proposal for standardization of optimized mycobacterial interspersed repetitive unit-variable-number tandem repeat typing of Mycobacterium tuberculosis. Journal of clinical microbiology. 2006 12;44(12):4498–510. . eng. 17005759 10.1128/JCM.01392-06 PMC1698431

[7] Allix-Beguec C, Wahl C, Hanekom M, Nikolayevskyy V, Drobniewski F, Maeda S, et al Proposal of a consensus set of hypervariable mycobacterial interspersed repetitive-unit-variable-number tandem-repeat loci for subtyping of Mycobacterium tuberculosis Beijing isolates. Journal of clinical microbiology. 2014 1;52(1):164–72. Pubmed Central PMCID: 3911419. 10.1128/JCM.02519-13 24172154 PMC3911419

[8] Luo T, Yang C, Pang Y, Zhao Y, Mei J, Gao Q. Development of a hierarchical variable-number tandem repeat typing scheme for Mycobacterium tuberculosis in China. PloS one. 2014;9(2):e89726 Pubmed Central PMCID: 3934936. 10.1371/journal.pone.0089726 24586989 PMC3934936

[9] de Beer JL, Kodmon C, van Ingen J, Supply P, van Soolingen D, Global Network for Molecular Surveillance of T. Second worldwide proficiency study on variable number of tandem repeats typing of Mycobacterium tuberculosis complex. The international journal of tuberculosis and lung disease: the official journal of the International Union against Tuberculosis and Lung Disease. 2014 5;18(5):594–600. . 24903798 10.5588/ijtld.13.0531

[10] de Beer JL, Kremer K, Kodmon C, Supply P, van Soolingen D, Global Network for the Molecular Surveillance of T. First worldwide proficiency study on variable-number tandem-repeat typing of Mycobacterium tuberculosis complex strains. Journal of clinical microbiology. 2012 3;50(3):662–9. Pubmed Central PMCID: 3295139. 10.1128/JCM.00607-11 22170917 PMC3295139

[11] Matsumoto T, Koshii Y, Sakane K, Murakawa T, Hirayama Y, Yoshida H, et al A novel approach to automated genotyping of Mycobacterium tuberculosis using a panel of 15 MIRU VNTRs. Journal of microbiological methods. 2013 6;93(3):239–41. 10.1016/j.mimet.2013.03.022 23566824

[12] Gauthier M, Bidault F, Mosnier A, Bablishvili N, Tukvadze N, Somphavong S, et al High-throughput mycobacterial interspersed repetitive-unit-variable-number tandem-repeat genotyping for Mycobacterium tuberculosis epidemiological studies. Journal of clinical microbiology. 2015 2;53(2):498–503. Pubmed Central PMCID: 4298502. 10.1128/JCM.01611-14 25428144 PMC4298502

[13] Supply P. Multilocus Variable Number Tandem Repeat Genotyping of Mycobacterium tuberculosis. Technical Guide. 2005.

[14] Brown TJ, Nikolayevskyy VN, Drobniewski FA. Typing Mycobacterium tuberculosis using variable number tandem repeat analysis. Methods Mol Biol. 2009;465:371–94. Epub 2009/01/01. eng. 10.1007/978-1-59745-207-6_25 20560063

[15] Velji P, Nikolayevskyy V, Brown T, Drobniewski F. Discriminatory ability of hypervariable variable number tandem repeat loci in population-based analysis of Mycobacterium tuberculosis strains, London, UK. Emerging infectious diseases. 2009 10;15(10):1609–16. Epub 2009/10/29. eng. 10.3201/eid1510.090463 19861054 PMC2866407

[16] Yokoyama E, Kishida K, Uchimura M, Ichinohe S. Comparison between agarose gel electrophoresis and capillary electrophoresis for variable numbers of tandem repeat typing of Mycobacterium tuberculosis. Journal of microbiological methods. 2006 6;65(3):425–31. . eng. 16219376 10.1016/j.mimet.2005.08.014

[17] Rosenblum BB, Oaks F, Menchen S, Johnson B. Improved single-strand DNA sizing accuracy in capillary electrophoresis. Nucleic Acids Res. 1997 10 1;25(19):3925–9. . Pubmed Central PMCID: 146964. 9380518 10.1093/nar/25.19.3925 PMC146964

[18] Gopaul KK, Brown TJ, Gibson AL, Yates MD, Drobniewski FA. Progression toward an improved DNA amplification-based typing technique in the study of Mycobacterium tuberculosis epidemiology. Journal of clinical microbiology. 2006;44(7):2492–8. 16825370 10.1128/JCM.01428-05 PMC1489471

[19] Nikolayevskyy V, Gopaul K, Balabanova Y, Brown T, Fedorin I, Drobniewski F. Differentiation of tuberculosis strains in a population with mainly Beijing-family strains. Emerging infectious diseases. 2006 9;12(9):1406–13. . eng. 17073090 10.3201/eid1209.041263 PMC3294723

[20] Bidovec-Stojkovic U, Seme K, Zolnir-Dovc M, Supply P. Prospective genotyping of Mycobacterium tuberculosis from fresh clinical samples. PloS one. 2014;9(10):e109547 Pubmed Central PMCID: 4196917. 10.1371/journal.pone.0109547 25313883 PMC4196917

[21] Viana Niero C, de HPE, van SD, Leao SC. Analysis of genetic polymorphisms affecting the four phospholipase C (plc) genes in Mycobacterium tuberculosis complex clinical isolates. Microbiology. 2004;150(Pt 4):967–78. 15073306 10.1099/mic.0.26778-0

[22] Faksri K, Drobniewski F, Nikolayevskyy V, Brown T, Prammananan T, Palittapongarnpim P, et al Genetic diversity of the Mycobacterium tuberculosis Beijing family based on IS6110, SNP, LSP and VNTR profiles from Thailand. Infection, genetics and evolution: journal of molecular epidemiology and evolutionary genetics in infectious diseases. 2011 7;11(5):1142–9. 10.1016/j.meegid.2011.04.007 21515409

